# A combination of baseline plasma immune markers can predict therapeutic response in multidrug resistant tuberculosis

**DOI:** 10.1371/journal.pone.0176660

**Published:** 2017-05-02

**Authors:** Selena Ferrian, Claudia Manca, Sugnet Lubbe, Francesca Conradie, Nazir Ismail, Gilla Kaplan, Clive M. Gray, Dorothy Fallows

**Affiliations:** 1 Division of Immunology, Institute of Infectious Disease and Molecular Medicine and National Health Laboratory Services, University of Cape Town, Cape Town, South Africa; 2 Public Health Research Institute, Rutgers University, Newark, New Jersey, United States of America; 3 Department of Statistical Sciences, University of Cape Town, Cape Town, South Africa; 4 Right to Care and the Clinical HIV Research Unit, University of the Witwatersrand, Johannesburg, South Africa; 5 Centre for Tuberculosis, National Institute for Communicable Diseases, Johannesburg, South Africa; 6 Bill & Melinda Gates Foundation, Seattle, Washington, United States of America; National Institute of Infectious Diseases, JAPAN

## Abstract

**Objective:**

To identify plasma markers predictive of therapeutic response in patients with multidrug resistant tuberculosis (MDR-TB).

**Methods:**

Fifty HIV-negative patients with active pulmonary MDR-TB were analysed for six soluble analytes in plasma at the time of initiating treatment (baseline) and over six months thereafter. Patients were identified as sputum culture positive or negative at baseline. Culture positive patients were further stratified by the median time to sputum culture conversion (SCC) as fast responders (< 76 days) or slow responders (≥ 76 days). Chest X-ray scores, body mass index, and sputum smear microscopy results were obtained at baseline.

**Results:**

Unsupervised hierarchical clustering revealed that baseline plasma levels of IP-10/CXCL10, VEGF-A, SAA and CRP could distinguish sputum culture and cavitation status of patients. Among patients who were culture positive at baseline, there were significant positive correlations between plasma levels of CRP, SAA, VEGF-A, sIL-2Rα/CD40, and IP-10 and delayed SCC. Using linear discriminant analysis (LDA) and Receiver Operating Curves (ROC), we showed that a combination of MCP-1/CCL2, IP-10, sIL-2Rα, SAA, CRP and AFB smear could distinguish fast from slow responders and were predictive of delayed SCC with high sensitivity and specificity.

**Conclusion:**

Plasma levels of specific chemokines and inflammatory markers measured before MDR-TB treatment are candidate predictive markers of delayed SCC. These findings require validation in a larger study.

## Introduction

The global emergence of multidrug resistant tuberculosis (MDR-TB) represents a growing public health issue. Of the estimated 9.6 million new TB cases which occurred in 2014, roughly 5% (480,000 cases) were due to infection by MDR *Mycobacterium tuberculosis* [1]. MDR is defined as resistance *in vitro* to isoniazid and rifampicin, the two most effective anti-TB drugs and the cornerstone of standard combination regimens. Treatment of MDR-TB requires drugs that are more toxic and less effective than first-line antibiotics, and chemotherapy must be administered for up to 2 years or longer. MDR-TB is associated with a poorer therapeutic outcome and elevated mortality rates compared to drug susceptible disease, and the lengthy regimens and high frequency of adverse events lead to greater problems with adherence for patients [2]. Research is underway to discover new chemotherapeutic agents and regimens, aimed to shorten the duration of treatment and improve outcome for both MDR and drug susceptible (DS) TB [3]. However, the conduct of clinical trials in the context of MDR-TB is both challenging and expensive, in part due to the lengthy follow-up necessary to establish treatment outcome and 2-year relapse rates [4]. Early markers predictive of clinical outcome can provide surrogate endpoints to accelerate the drug discovery pipeline and/or help to stratify patients for clinical trials. In addition, such markers could identify cases at higher risk of treatment failure for intensive monitoring and may help to inform therapeutic decisions for improved outcome.

Sputum culture conversion (SCC), evaluated at 2 months of therapy, is the only TB biomarker currently used as an early microbiological endpoint in phase II clinical trials, based on its demonstrated ability to predict clinical outcome and relapse in DS-TB [5, 6]. Among MDR-TB patients, 2-month SCC has shown a strong association with treatment outcome [7, 8]. In contrast, sputum acid-fast bacilli (AFB) smear, which provides an indication of bacillary load and is often used to monitor TB patients in resource-poor settings, does not distinguish live and dead organisms and is not predictive of outcome or relapse. Thus, there is an urgent need to identify early biomarkers of treatment efficacy that could be developed as rapid point-of-care tests for patient stratification in clinical management and trial settings. A number of immunological markers, measured in blood at the start of treatment, have shown promising results as prognostic markers of clinical severity and/or predictors of microbiological outcome in TB patients [9–11]. However, the majority of these studies have been conducted in patients with DS-TB, and the results may not be generalizable to MDR-TB cases. Several reports have described lower levels of Th1 cytokines [12–14] and higher numbers of T regulatory (Treg) cells [15–17] in peripheral blood from MDR, compared to DS, TB patients. The trend towards greater immune suppression in MDR-TB may be related to a longer duration of disease and more extensive lung damage, as suggested by the recovery of cell mediated immunity following pulmonary resection to remove severely diseased tissue [18]. Nonetheless, the findings support the need for specific evaluation of biomarkers in MDR-TB.

We previously described a population of mycobacteria-specific CD4+ T cell that predicts the microbiological outcome of treatment in MDR-TB patients [19]. In the present study, we investigated immune markers in plasma, sampled before and during treatment, to identify promising candidates that were predictive of microbiological outcome in 50 HIV-negative MDR-TB patients. The following markers were analyzed as potential biomarkers of response to treatment, based on previously reported associations in DS-TB patients: interferon gamma-induced protein 10 (IP-10)/CXCL10, vascular endothelial growth factor A (VEGF-A), soluble interleukin-2 receptor alpha (sIL-2Rα)/CD25, C-reactive protein (CRP), serum amyloid A (SAA) and monocyte chemoattractant protein 1 (MCP-1)/CCL2 [11, 20–23].

## Materials and methods

### Study participants

A total of 50 patients initiating treatment for MDR-TB at Sizwe Hospital in Johannesburg, South Africa were enrolled in the study. The patients had a median age of 36 years (IQR:22–49 years), 43% were female and all were HIV-negative. Diagnosis of MDR-TB was based on sputum culture (Bactec MGIT 960, Beckton Dickinson, Baltimore, MD) and phenotypic drug susceptibility testing (DST; Bactec MGIT 960) and/or genotypic resistance by PCR (Line Probe Assay, Hain Lifesciences). Baseline (enrollment) clinical and microbiological characteristics are summarized in Table 1. Nine study participants were sputum culture negative at the time of enrollment; however, all of these patients were sputum culture positive at a median of 48 days (IQR: 16.5–76.0) prior to baseline, suggesting that their infections were partially responsive to first-line drugs. Eight of the culture negative patients (88.9%) had received prior TB treatment, and all were sputum smear negative. Of the 41 patients who were sputum culture positive at baseline, 38 (92.7%) had a prior history of TB treatment (Table 1). A wide diversity in body mass index (BMI) was noted overall (median 19.0; IQR: 17.7–22.8), with 20 patients (45%) classified as underweight (<18.5), 17 (39%) as normal BMI (18.5–24.9), six (14%) as overweight (25.0–29.9) and one patient classified as obese (>30), based on WHO criteria.

**Table 1:** Baseline clinical and microbiological characteristics of MDR-TB patients.

	Culture Negative (n = 9)		Culture Positive Fast Responder (n = 19)		Culture Positive Slow Responder (n = 22)		*P*^§^
Median Age, years (IQR)	47	(25.5–59)	37	(26–45)	27	(22–49)	
Female	3	(37.5%)	10	(52.6%)	8	(36.4%)	0.2325
Prior TB Treatment	8	(100%)	16	(84.2%)	22	(100%)	0.0909
Sputum AFB Smear							
0 or scanty	8	(100%)	8	(44.4%)	4	(19.0%)	0.0707
1+	0	-	4	(22.2%)	3	(14.3%)	
2+	0	-	3	(16.7%)	2	(9.5%)	
3+	0	-	3	(16.7%)	12	(57.1%)	
Lung involvement^a^							
None	4	(50.0%)	1	(5.6%)	0	-	0.4543
Unilateral	1	(12.5%)	3	(16.7%)	6	(30.0%)	
Bilateral	3	(37.5%)	14	(77.8%)	14	(70.0%)	
Cavitation^a^							
No	8	(100.0%)	6	(33.3%)	2	(10.0%)	0.0860
Yes	0	-	12	(66.7%)	18	(90.0%)	
BMI							
< 18.5	3	(37.5%	5	(27.8%)	12	66.7%)	0.0888
18.5–24.9	3	(37.5%)	9	(50.0%)	5	27.8%)	
25–29.9	1	(12.5%)	4	(22.2%)	1	5.6%)	
≥ 30	1	(12.5%)	0	-	0	-	
FQN resistant	-		3	(15.8%)	5	(22.7%)	0.4378
PZA resistant	-		12	(85.7%)	13	(68.4%)	0.2340
Avg # effective drugs							
<4	-		18	(94.7%)	16	(72.7%)	0.1335
4 to <5	-		1	(5.3%)	5	(22.7%)	
5 to <6	-		0	-	1	(4.5%)	
≥6	-		0		0	-	
Avg # potentially effective drugs							
<4	-		1	(5.3%)	0	-	0.3724
4 to <5	-		4	(21.1%)	3	(13.6%)	
5 to <6	-		11	(57.9%)	11	(50.0%)	
≥6	-		3	(15.8%)	8	(36.4%)	
Months of PZA+FQN+CFZ							
<6	-		3	(15.8%)	3	(13.6%)	0.4778
6 to 9	-		11	(57.9%)	9	(40.9%)	
>9	-		5	(26.3%)	10	(45.5%)	
							

AFB: Acid Fast Bacilli. BMI: Body mass index.

^a^ Based on chest X-ray reviews.

§ Fast vs Slow Responders

### Drug susceptibility testing and bacteriological response to treatment

Sputum culture and AFB smear microscopy were performed at the time of admission and monthly thereafter, as part of routine monitoring. Sputum culture conversion (SCC) was defined as two consecutive TB culture negative results, separated by at least 30 days, with no subsequent culture positive results. Time to SCC was defined as the interval between the date of treatment initiation and the date of collection of the sputum specimen that yielded the first negative culture result. Patients who were sputum culture positive at enrollment were classified, based on the median number of days to achieve SCC in this cohort, as either slow responders (≥ 76 days) or fast responders (< 76 days) for analysis (Table 1).

Drug susceptibility testing (DST) (Bactec MGIT 960) was performed for all culture positive MDR-TB patients within 30 days of starting treatment. DST results, obtained from patient records, were available for the following drugs: isoniazid, rifampin, ethambutol, ethionamide, streptomycin, kanamycin and ofloxacin. As pyrazinamide DST is not routinely performed, these results were only available from 33 patients (14/19 fast; 19/22 slow responders). DST was not done for any other drugs, including clofazimine. Effective and potentially effective drugs in individual patient regimens were defined as those with known susceptibility only and those with no known resistance, respectively [24]. DST results and TB drug regimens of individual patients are shown in S1 Table.

### Assessment of disease severity by chest X-ray

Disease severity was evaluated based on chest X-ray, performed at the time of admission to hospital and scored for extent of lung involvement and cavitation. Chest X-ray reviews were obtained from patient records. Cavitary lesions were present in 30 (65%) of the 46 patients with available chest X-ray reviews, of whom 22 (73.3%) had bilateral lung involvement (Table 1). Of the 8 sputum culture negative patients with chest X-ray reviews available, none showed signs of cavitary disease.

### Patient informed consent

All study participants gave written, informed consent for the study, which was approved by the Research Ethics Committees of the University of the Witwatersrand and University of Cape Town and the Institutional Review Board of Rutgers University (Pro0120090189).

### Luminex multiplex immunoassay

Blood samples were collected on the day after patients started MDR-TB therapy and were enrolled into the study and thereafter at 2, 4, and 6 months of treatment. Peripheral blood was obtained in sodium heparin Vacutainer tubes (BD Biosciences, San Diego, CA); plasma was prepared within 45 minutes of collection and stored at −80°C for later analysis. Stored plasma samples were rapidly thawed and analyzed for the following markers: sIL-2Rα/CD25, VEGF-A, IP-10/CXCL10, SAA, CRP, and MCP-1/CCL2. Plasma samples were diluted (1:1000 for CRP, 1:500 for SAA, 1:5 for all other analyses) and concentrations were determined using multiplex immunoassays, according to the manufacturer’s instructions (ProcartaPlex Human kits, eBioscience, San Diego, CA), then read on a luminometer (Bioplex 200, BioRad, Hercules, CA). The reagent kits include analytes of known concentration which are used to generate a standard curve. The unit automatically determines a regression equation for the curve and calculates the concentrations of analyte in each sample. Concentration values outside the range of detection are automatically flagged and not displayed as a number.

### Statistical analysis

Distribution of variables was assessed by Shapiro-Wilk and D’Agostino & Pearson tests. Heat map and clustering of significant differences were analyzed using Qlucore Omics Explorer 3.1 with interface to R (Qlucore AB, Lund, Sweden). Analyte levels were log _2_ converted and standardized by subtraction of its mean value and division by its standard deviation across all samples. For concentrations that were outside the range of detection, the lowest detected level (LDL) or highest detected level (HDL) values were used to replace the out-of-range values prior to log_2_ conversion. To identify markers with expression levels that differed at baseline and over time between unpaired groups, the Mann-Whitney U test was used. All values were corrected for false discovery rate (FDR) using a q-value of ≤ 0.05. To analyze longitudinal changes in parameters during follow-up, the Wilcoxon signed rank test was performed. The relationship between the concentration of baseline plasma analytes and time to SCC was assessed by Pearson or Spearman rank correlations. Receiver Operating Characteristic (ROC) analysis was used to assess the predictive nature of each analyte with time to SCC. Results were considered significant if the 95% confidence interval (CI) of the area under the curve (AUC) exceeded 0.70. All possible combinations of host analytes were evaluated using linear discriminant analysis (LDA) to find the subset of analytes which best predicted fast from slow responders. Fisher’s exact test was used to compare drug resistance profiles and treatment regimens between the slow and fast responder groups.

## Results

### Associations between clinical characteristics and microbiology

When we examined the associations between clinical and microbiological data (Table 1), a strong association existed between the extent of lung pathology, based on chest X-ray scores, and sputum smear AFB at baseline (Spearman’s correlation coefficient r = 0.4811, p = 0.0008), where advanced radiological findings were positively correlated with higher smear grades. Likewise, patients who were culture positive at baseline were more likely to have pulmonary cavities than patients who were culture negative (p = 0.0001). Baseline chest X-ray scores also positively correlated with the time to SCC (Spearman’s correlation coefficient r = 0.5383, p = 0.0002), and there was a strong association between time to SCC and baseline smear microscopy grades (Spearman’s correlation coefficient r = 0.7293, p<0.0001). Collectively, these data show that disease severity (as indicated by chest X-ray scores) was directly associated with bacillary load.

### Plasma levels of analytes reflect sputum culture status

**Fig 1:**
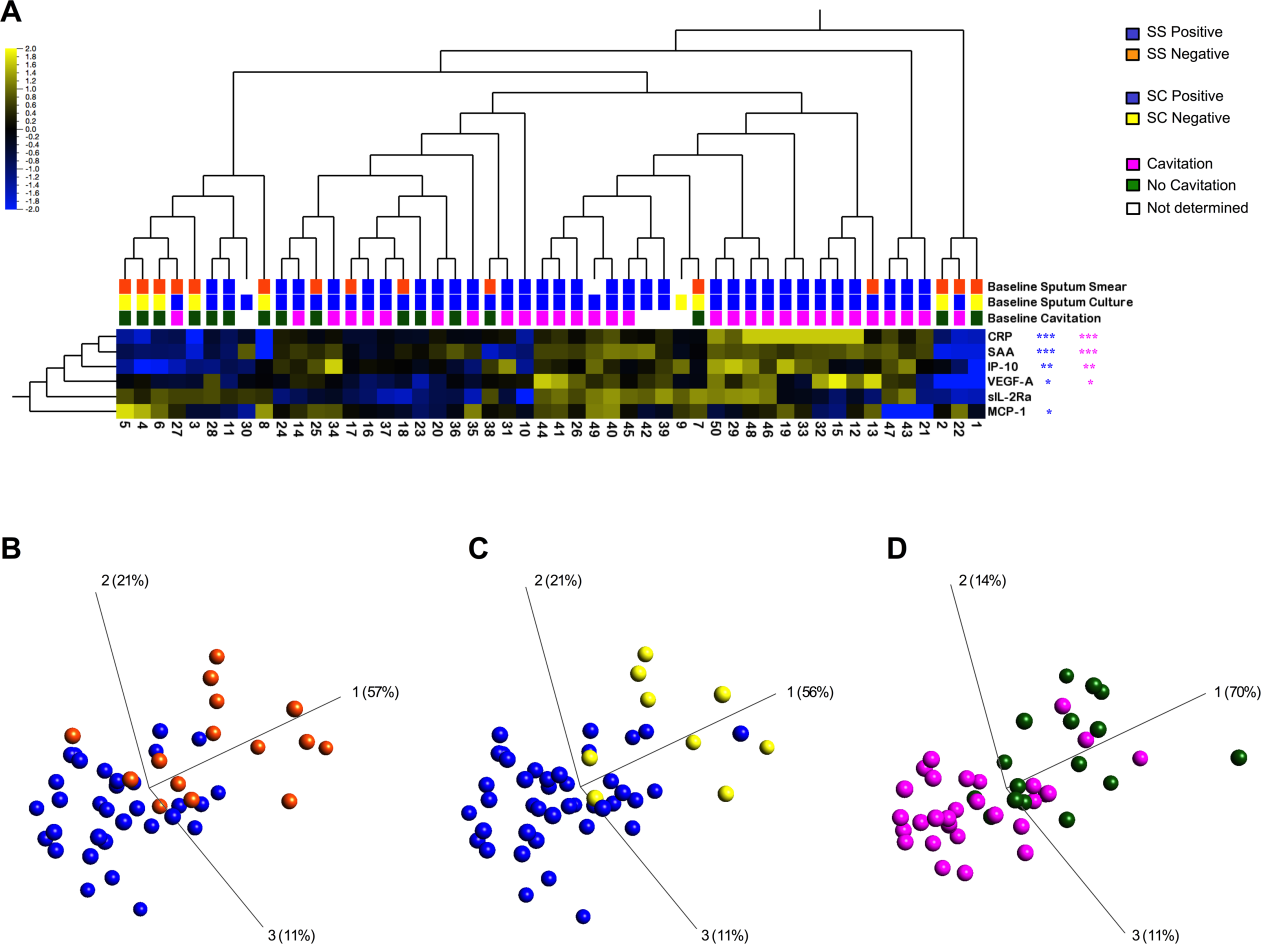
Baseline levels of plasma markers. (A) Two-dimensional unsupervised hierarchical clustering of baseline analyte profiles in 50 patients, characterized by sputum smear (SS) and sputum culture (SC) status and cavitary vs non-cavitary disease. Normalized and log2 transformed values of analyte levels are indicated by the color scale, where yellow and blue represent expression levels above and below the median, respectively. Three-dimensional plots of principal component analysis (PCA) of (B) SS negative (orange) and SS positive (blue); (C) SC negative (yellow) and SC positive (blue); (D) cavitary (pink) and non-cavitary disease (green). Statistical comparisons using non-parametric Mann-Whitney U test were corrected for multiple comparisons through a false discovery rate (FDR) step down procedure (*: q<0.05, **: q<0.01, ***: q<0.001).

We first interrogated how levels of plasma markers clustered with baseline sputum AFB smear and culture status. Fig 1A shows unsupervised hierarchical clustering of the 6 analytes at baseline for both sputum smear and culture. After correcting for FDR (q), we found significantly higher levels of CRP (q = 5.8355x10^-5^), SAA (q = 0.0001), IP-10 (q = 0.0038) and VEGF-A (q = 0.0224) in patients who were smear positive compared to smear negative. The inverse was found for MCP-1 levels, which were lower in smear positive cases (q = 0.0153). No significant differences were found for sIL-2Rα levels, although there was a trend towards lower levels in smear positive compared to smear negative cases (q = 0.2921). The distinct grouping of these plasma analytes with sputum smear status is reflected in the principal component analysis (PCA) plot (Fig 1B). Similarly, higher levels of CRP (q = 0.0005), SAA (q = 0.0005), IP-10 (q = 0.0086), and VEGF-A (q = 0.0368) and lower levels of MCP-1 (q = 0.0406) were found in plasma from TB culture positive compared to culture negative patients (Fig 1A), and these two patient groups were distinguished by the markers (Fig 1C).

As sputum culture positivity was strongly associated with cavitation (Fig 1A), it was not surprising to find a similar pattern of soluble markers co-clustered with both cavitation and baseline culture status. Higher levels of CRP (q = 0.0003), SAA (q = 0.0007), IP-10 (q = 0.0028) and VEGF-A (q = 0.0203) were associated with lung cavitation, while MCP-1 and sIL-2Rα showed no significant association. Fig 1D shows the PCA plot reflecting this distinct grouping between soluble markers and cavitation. Thus, levels of plasma IP-10, VEGF-A, SAA and CRP were able to discriminate between sputum culture positive and negative cases and between the presence and absence of lung cavitation at baseline.

### Baseline soluble plasma analytes as predictors of delayed sputum culture conversion (SCC)

**Fig 2:**
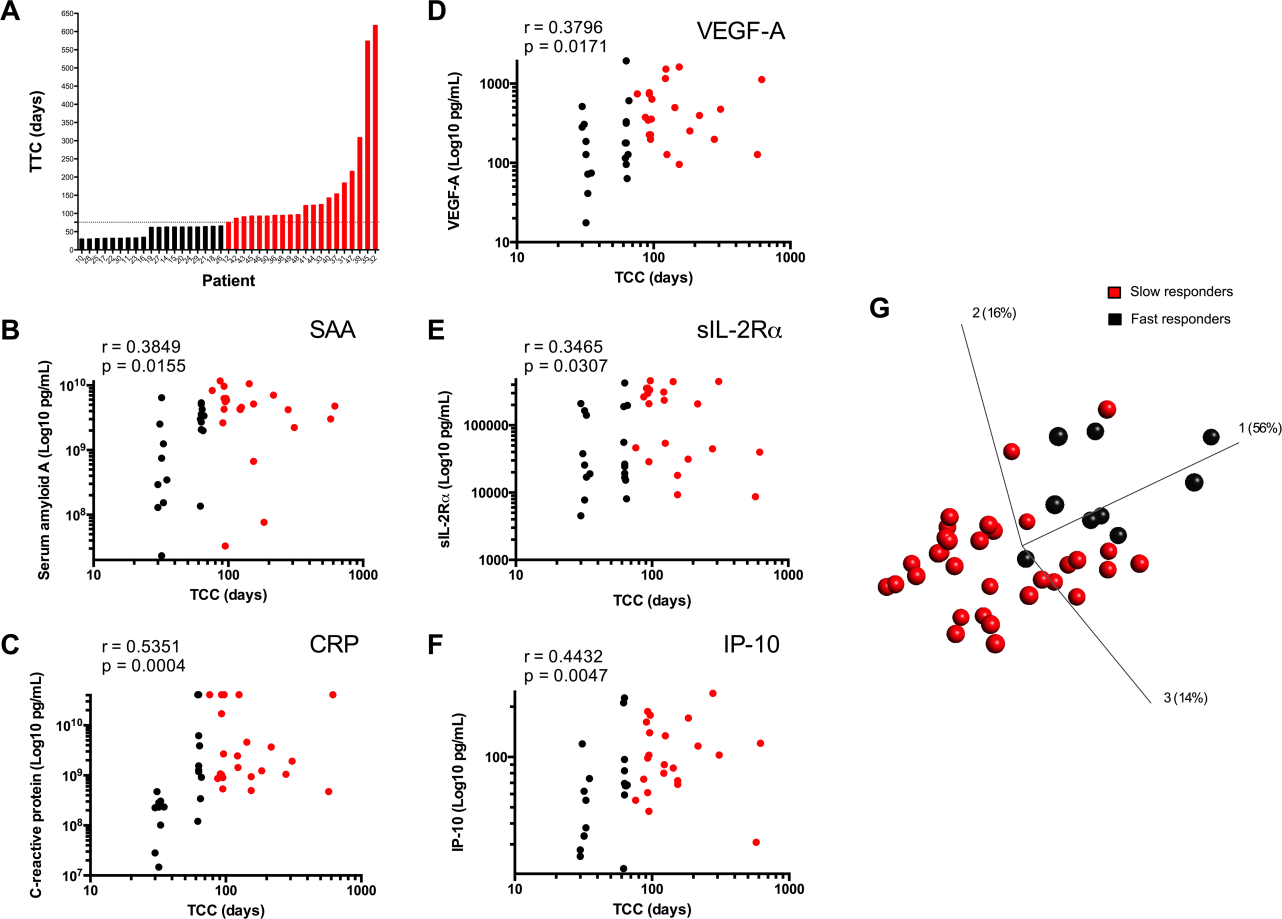
Expression of plasma markers in fast and slow responders. (A) Distribution of time to culture conversion (TCC) in study cohort; (B–F) Correlation between baseline levels of individual plasma markers and TCC, shown as slow (red) or fast (black) responders. (G) Principal component analysis (PCA) plot of slow (red) and fast (black) responders, analyzed as above.

A wide distribution in days to achieve SCC was noted among the MDR-TB patients who were sputum culture positive at baseline (Fig 2A). The median plasma levels of all markers, measured at baseline, were significantly different between fast and slow responders (Table 2). Moreover, there were significant positive correlations between delayed SCC and baseline levels of SAA, CRP, VEGF-A, sIL-2Rα and IP-10 (Fig 2B, 2C, 2D, 2E and 2F, respectively), while the combination of these markers showed a trend towards distinguishing fast and slow responder groups (Fig 2G). There was no significant association of MCP-1 with SCC (not shown).

**Table 2:** Baseline plasma concentrations of analytes in slow and fast responders.

Host analyte	Slow responders (n = 22) Median (IQR) pg/mL	Fast responders (n = 19) Median (IQR) pg/mL	*p*-value
VEGF-A	386.1 (220−749)	179 (74−317)	0.0026
SAA	4.7x10^9^ (2.9x10^9^−6.5x10^9^)	2x10^9^ (0.3x10^9^−3.6x10^9^)	0.0015
sIL-2Rα	2.2x10^5^ (0.38x10^5^−3.5x10^5^)	0.25x10^5^ (0.17x10^5^−1.6x10^5^)	0.0041
CRP	1.7x10^9^ (0.9x10^9^−23x10^9^)	0.34x10^9^ (0.2x10^9^−15x10^9^)	0.0063
IP-10	100 (71−145)	67 (34−83)	0.0091
MCP-1	122 (80−188)	83 (59−139)	0.1749

**Fig 3:**
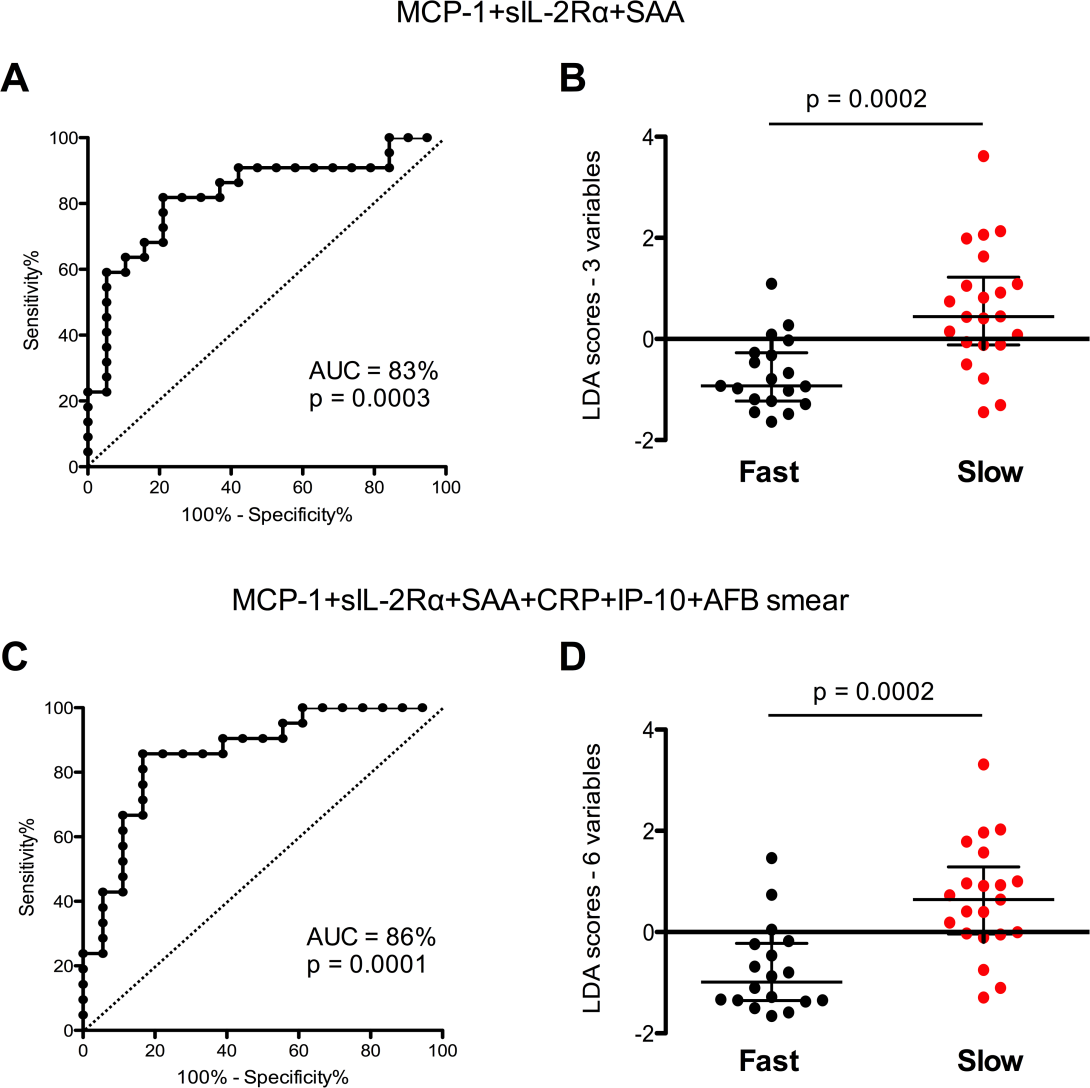
Plasma markers as predictors of fast vs slow response to treatment. Receiver Operating Characteristic (ROC) curve analysis and baseline LDA scores of (A and B) the optimal combination of plasma markers, and (C and D) the optimal combination of markers plus clinical data (sputum smear). Horizontal bars indicate median and interquartile range. Statistical analyses between unpaired groups were performed using non-parametric Wilcoxon paired tests. Differences between groups were assessed by Mann-Whitney U test. P<0.05 was considered significant.

We next asked whether any of the analytes could be useful predictors of delayed SCC. Baseline levels of single markers showed significant area under the curve (AUC), although with variably low sensitivity or specificity (S1 Table). However, when plasma levels of MCP-1 (CCL2), sIL-2Rα and SAA were combined, there was a significant area under the curve (AUC) of 83% (p = 0.0003) with 82% sensitivity and 79% specificity, and an optimal misclassification error rate of 19.5% (Fig 3A). A combination of MCP-1 (CCL2), IP-10 (CXCL10), sIL-2Rα, SAA and CRP, together with baseline smear status, yielded an AUC of 86% (p = 0.0001) with 86% sensitivity and 83% specificity, and an optimal misclassification error rate of 15.3% (Fig 3C). Moreover, slow responders had significantly greater LDA scores for both the 3-variable and the 6-variable combinations (Fig 3B and 3D), indicating that higher levels of these markers at the start of treatment were associated with delayed time to bacillary clearance.

**Fig 4:**
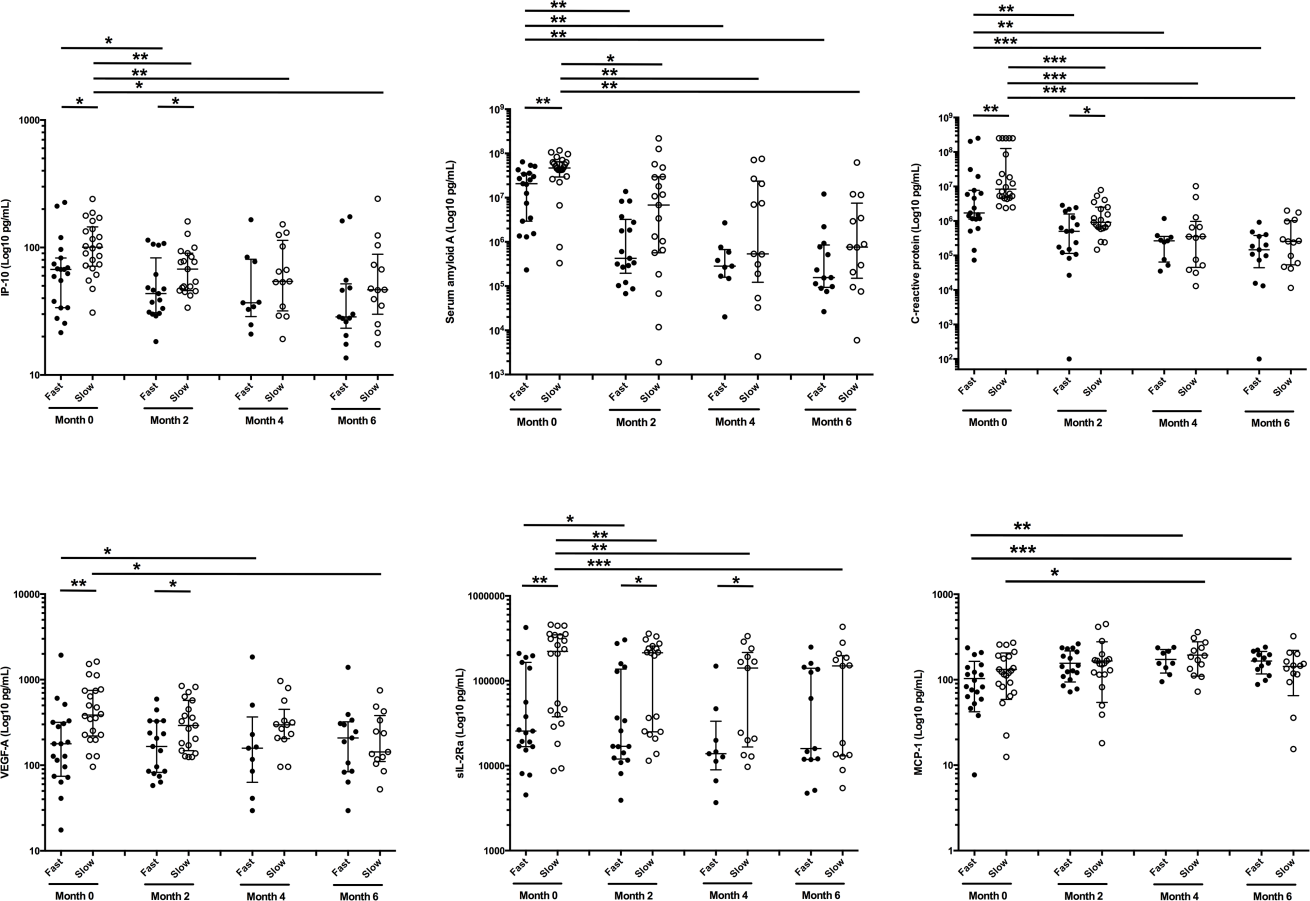
Longitudinal assessment of plasma analyte expression levels over therapy in slow and fast responders. The concentration of each analyte is shown at baseline (0), 2, 4 and 6 months after initiating an 18-month treatment regimen for MDR-TB in fast (solid circles) versus slow (open circles) responders. Results are expressed as Log_10_ pg/mL of plasma. Horizontal bars indicate median and interquartile range. Statistical analyses over time were performed using non-parametric Wilcoxon paired test. Differences between unpaired groups were assessed by Mann Whitney U test or parametric unpaired Welch t test. P<0.05 was considered significant.

When we examined the impact of treatment on these plasma analytes over 6 months of chemotherapy, both fast and slow responders showed significant reductions in levels of IP-10, SAA, CRP and sIL-2Rα at month 2, with a further decline of SAA in slow responders to month 4 (Fig 4). There was a downward trend in plasma levels of these four markers over time in slow responders, but in fast responders only SAA and CRP showed a decline over 6 months. Plasma levels of sIL-2Rα remained significantly higher in slow, compared to fast, responders until month 6. VEGF-A levels were higher in slow responders over the first two months and showed a significant drop from baseline levels to month 6 (p = 0.0266). Thus, effective treatment of MDR-TB resulted in reduced levels of the markers over time, with some mediators responding at different rates between fast and slow responders. As a result, the predictive nature of these analytes to time to SCC was lost by month 2 of chemotherapy, most notably for SAA and CRP (data not shown).

MDR-TB regimens including a greater number of effective drugs (i.e. no known resistance) have been correlated with improved response to therapy [24], while fluoroquinolones (FQN), pyrazinamide (PZA) and clofazimine (CFZ) are known to accelerate bacillary clearance. To examine the possibility that treatment differences between the two patient groups may have been responsible for the observed differential SCC, we compared drug regimens for fast and slow responders (Table 1). There were no significant differences in the average number of effective or potentially effective drugs (p = 0.1335; p = 0.3724, respectively) nor in the cumulative months of drugs known to accelerate bacillary clearance, pyrazinamide, clofazimine and fluoroquinolone (p = 0.4778), included in the regimens between the fast and slow responders. Whatever the mechanism accounting for the rapidity of bacterial clearance, baseline plasma analytes were able to predict the rate of this occurring.

## Discussion

The goal of this study was to identify plasma markers that are predictive of response to treatment in MDR-TB patients. Although similar studies have been carried out in patients initiating conventional treatment for DS-TB [20, 21, 25], there have been few reports on early markers of response to treatment among MDR-TB patients. In this study, we examined the potential of six plasma markers, measured at the start of second-line drug treatment to predict the time to sputum culture conversion (SCC) in MDR-TB patients.

CRP and SAA are acute phase proteins produced by the liver and released during inflammation, functioning as opsonins and in recruitment of cells to inflammatory sites, respectively [26]. Serum CRP has demonstrated value in diagnosis of active TB in both HIV-positive [27–29] and HIV-negative patients [30, 31]. Positive correlations have been noted between circulating CRP levels and cavitation/impaired lung function [32–35] and sputum culture status [36]. High CRP levels have been associated with delayed SCC [10, 37], and a decline in CRP was correlated with clinical response to treatment, although CRP alone is a poor predictor of outcome due to its low specificity [38]. Serum levels of SAA were similarly elevated in TB patients compared to healthy controls [30, 31] and have been associated with sputum culture status and response to TB treatment [36, 39]. Consistent with these reports, baseline levels of CRP and SAA correlated with both cavitation and sputum culture status in our study cohort, and both acute phase markers declined with treatment.

VEGF is a key mediator of angiogenesis and lymphangiogenesis in granulomas and plays an important role in TB pathogenesis [40]. In the present study, we found a significant positive correlation between baseline levels of VEGF-A and delayed response to MDR-TB treatment. In addition, VEGF-A levels were positively associated with both cavitation and positive TB culture, suggesting that this marker may be a surrogate measure of disease severity. Elevated levels of VEGF have been found in serum and saliva from patients with DS-TB compared to healthy controls or individuals with latent TB infection (LTBI) [30, 41–43] and have been positively correlated with bilateral and/or cavitary TB and sputum AFB smear grade, as a measure of bacillary burden [40], similar to our observations. In a recent study, treatment with anti-VEGF antibody was found to normalize vascularization, decrease hypoxia and reduce granuloma size in a rabbit model of pulmonary TB, supporting a functional role for this molecule in TB pathogenesis [44]. In our MDR cohort, plasma levels of VEGF-A did not decline in fast responders over the first 6 months of treatment, while slow responders showed a significant drop in levels only after 5 months of chemotherapy. In contrast, several studies have reported a decrease in circulating VEGF levels in association with successful treatment of DS-TB [11, 40, 41, 45] and tubercular meningitis [46]. Persistently elevated VEGF-A in our MDR-TB cohort may be an indication of delayed pulmonary recovery despite microbiological response to treatment. However, follow up chest X-rays were not available, so we are not able to confirm this possibility.

Soluble IL-2Rα is involved in T cell homeostasis, and elevated levels in blood are considered to be an indication of sustained immune activation [47]. In our cohort, higher levels of sIL-2Rα in plasma at baseline were significantly associated with delayed SCC. Similar results were described from a recent study of patients with DS-TB, although the trend of higher sIL-2Rα levels in slow versus fast responders did not reach significance [21]. Other studies have reported elevated serum levels of sIL-2Rα in TB patients compared to healthy controls, which declined with successful treatment [48–53]. In contrast, the patients in our MDR cohort showed persistently high plasma levels of sIL-2Rα over 6 months of therapy, which may reflect continuing immune activation despite bacillary clearance. Interestingly, sIL-2Rα has been shown to impair T cell proliferation and to induce differentiation of Treg cells in vitro [47]. Although we did not examine T cell subsets in this study, it is possible that the persistently high sIL-2Rα levels in blood observed may have contributed to elevated Treg cells in these MDR-TB patients, as has been previously reported [15, 16].

Serum levels of the chemokine IP-10 (CXCL10) have demonstrated diagnostic value in differentiating between active TB and LTBI and to decline with treatment [11, 20, 54, 55]. In contrast to these reports, despite significant differences in IP-10 levels between fast and slow responders in our study, baseline levels of IP-10 alone showed relatively low sensitivity as a predictor of response to treatment. While it is difficult to make comparisons between absolute levels of plasma markers in different studies, it is interesting to note that IP-10 levels in plasma from our MDR patients described here were considerably lower than those reported in similar studies conducted in DS-TB patients [11, 20, 30, 54]. One possible explanation for these discrepancies may be related to the suppression of Th1 responses previously reported in association with MDR-TB [12, 13, 56, 57]. A large proportion (92%) of the patients in our study had a previous history of treatment for TB. Repeated exposure to TB antigens in re-treatment cases may be responsible for the characteristics of immune suppression seen in MDR-TB patients, who often have a prior history of disease. This idea is supported by a recent study of DS patients showing reduced responsiveness to TB antigens of PBMCs from re-treatment as compared to new cases, including lower production of IP-10 [58]. Collectively, the differential kinetics of decline in these plasma markers between slow and fast response to drug regimens resulted in loss of predicting time to SCC when analyzed after 2 months of treatment.

It is important to note that the present study is a pilot involving a small number of patients, which requires validation through a larger cohort study. Other limitations include the lack of DST results for clofazimine and pyrazinamide.

In conclusion, this study demonstrates that a combination of chemokines and markers of acute inflammation can be used as predictors of microbiological outcome in MDR-TB patients and have utility as potential early indicators in clinical trials and in guiding therapy for patients who may be at elevated risk of TB treatment failure.

## Supporting information

S1 Table Fast and slow responder DST results and treatment regimens. (DOCX)

S2 Table Area-under-the-curve (AUC) of each analyte as a predictor of time to culture conversion. (DOCX)
