# Supplementary material for: A combination of baseline plasma immune markers can predict therapeutic response in multidrug resistant tuberculosis
Source: PLoS One. 2017 May 2;12(5):e0176660. doi: 10.1371/journal.pone.0176660 (PMC5413057; doi:10.1371/journal.pone.0176660)
Supplement: S1 Table — (DOCX) [file pone.0176660.s001.docx]

**S1 Table: Fast and slow responder DST results and treatment regimens**

| **PID** | **TCC** | **EMB** | **PZA** | **KAN** | **OFX** | **Eth** | **Intensive Regimen (4 months)** | **Continuation Regimen** |
| --- | --- | --- | --- | --- | --- | --- | --- | --- |
| 10 | F | S |  | S | S | R | Z Km Ox Et Tz | Z Km Ox Et Tz |
| 11 | F | R | R | S | S | R | Z Km Mx Tz Cz PAS Cla | Z Km Mx Tz Cz PAS Cla |
| 12 | F | R |  | S | S | R | E Z Km Ox Et Tz H Cz Az | E Z Km Ox Et Tz H Cz Az |
| 13 | F |  | S |  | S |  | E Z Km Mx Et Tz | E Z Km Mx Et Tz |
| 14 | F | R |  | S | R | R | Z Km Mx Tz H PAS Az | Z Mx Tz H PAS Az |
| 15 | F |  |  |  | *R* |  | E Z Km Mx Et Tz | E Z Km Mx Et Tz |
| 16 | F | S |  | S | S | R | E Z Km Mx Et Tz | E Z Km Mx Et Tz |
| 17 | F | R | R | R | S | R | E Z Ox Et Tz Cp | E Z Km Ox Et Tz Cz PAS Cla |
| 18 | F |  | S |  | S |  | E Z Km Ox Et Tz | E Z Km Ox Et Tz |
| 19 | F | R |  | S | S | S | E Z Km Ox Et Tz | Z Km Ox Et Tz |
| 20 | F | S | R | S | S | R | Z Km Mx Et Tz PAS | Z Km Mx Et Tz H Cz PAS |
| 21 | F | R | R | S | S | S | E Z Km Ox Et Tz PAS | E Z Km Ox Et Tz PAS |
| 22 | F |  | R |  | S |  | E Z Km Ox Et Tz | E Z Km Ox Et Tz |
| 23 | F | S | R | S | S | S | E Z Km Ox Et Tz | E Z Km Ox Et Tz |
| 24 | F | R | R | S | S | S | E Z Km Mx Et Tz Cz PAS | E Km Mx Et Tz Cz PAS |
| 25 | F |  | R |  | S |  | E Z Km Mx Et Tz | E Z Km Mx Et Tz |
| 26 | F | R | R | S | S | S | Z Km Ox Et Tz | Z Km Ox Et Tz |
| 27 | F | R | R | S | R | R | E Z Km Mx Tz H Cz PAS Cla | E Z Km Mx Tz H Cz PAS Cla |
| 28 | F | R | R | S | S | R | E Z Km Ox Et Tz Cz | E Z Km Ox Et Tz Cz |
| 29 | S | S | S | S | S | R | E Z Km Ox Et Tz Cla | E Z Km Ox Et Tz Cla |
| 30 | S | R | S | S | S | S | E Z Km Mx Et Tz Cz PAS | E Z Km Mx Et Tz Cz PAS |
| 31 | S | R | R | S | S | R | E Z Km Mx Et Tz Cz PAS | E Z Km Mx Et Tz Cz PAS |
| 32 | S |  |  |  | S |  | E Z Km Mx Et Tz | E Z Km Mx Et Tz |
| 33 | S | R | R | S | S | R | Z Km Mx Tz Cz PAS | Z Km Mx Tz Cz PAS |
| 34 | S | R |  | S | S |  | E Z Km Mx Et Tz | E Z Km Mx Et Tz |
| 35 | S | R |  | S | S | R | Z Km Mx Tz Cz PAS Cla | Z Km Mx Tz Cz PAS Cla |
| 36 | S | R | S | S | S | S | Z Km Mx Et Tz PAS Az | Z Km Mx Et Tz PAS Az |
| 37 | S | S | R | S | S |  | E Z Km Mx Et Tz | E Z Km Mx Et Tz |
| 38 | S | R | R | S | R | R | Z Km Mx Tz Cz PAS Az Am | Z Km Mx Tz Cz PAS Az Am |
| 39 | S | R | R | S | S |  | E Z Km Mx Et Tz Cz | E Z Km Mx Et Tz Cz |
| 40 | S | R | R | S | R | S | E Z Km Mx Et Tz Cz PAS Lz | E Z Km Mx Et Tz Cz PAS Lz |
| 41 | S | R | R | S | S | R | E Z Km Ox Et Tz PAS | E Z Km Ox Et Tz PAS |
| 42 | S | R | R | S | S | R | E Z Km Mx Et Tz H Cz PAS Cla | E Z Km Mx Et Tz H Cz PAS Cla |
| 43 | S | S | S | S | S | S | E Z Km Ox Et Tz | Z Km Ox Et Tz |
| 44 | S | R | R | S | R | R | Z Km Tz H Cz Cla | Z Km Tz H Cz Cla |
| 45 | S | R | S | S | S | S | E Z Km Ox Et Tz R PAS | E Z Km Ox Et Tz R PAS |
| 46 | S | R | S | S | S | S | E Z Km Mx Et Tz | E Z Km Mx Et Tz |
| 47 | S | R | R | R | R | R | E Z Mx Tz H Cz PAS Az Cp | E Z Mx Tz H Cz PAS Az Cp |
| 48 | S | R | R | R | S | R | Z Mx H Cz Cla Cp | Z Mx H Cz Cla Cp |
| 49 | S | R | R | S | S | R | E Z Km Ox Et Tz PAS Cla | E Z Km Ox Et Tz PAS Cla |
| 50 | S | R | R | R | R | R | E Mx Et Tz H Cz PAS Cla Cp | E Mx Et Tz H Cz PAS Cla Cp |
