# Supplementary material for: A combination of baseline plasma immune markers can predict therapeutic response in multidrug resistant tuberculosis
Source: PLoS One. 2017 May 2;12(5):e0176660. doi: 10.1371/journal.pone.0176660 (PMC5413057; doi:10.1371/journal.pone.0176660)
Supplement: S2 Table — (DOCX) [file pone.0176660.s002.docx]

**S2 Table: Area-under-the-curve (AUC) of each analyte as a predictor of time to culture conversion**

| **Host analyte** | **AUC (95% CI)** | ***p*-value** | **Sensitivity % (95% CI)** | **Specificity % (95% CI)** |
| --- | --- | --- | --- | --- |
| VEGF-A | 0.77 (0.62−0.92) | 0.0033 | 64 (41-83) | 84 (60-97) |
| SAA | 0.77 (0.61−0.92) | 0.0037 | 73 (50-89) | 79 (54-94) |
| sIL-2Rα | 0.76 (0.61−0.91) | 0.0048 | 59 (36-79) | 89 (67-99) |
| CRP | 0.75 (0.59−0.91) | 0.0071 | 95 (77-100) | 58 (34-80) |
| IP-10 | 0.74 (0.58−0.9) | 0.0101 | 63 (41-83) | 79 (54-94) |
| MCP-1 | 0.63 (0.46−0.80) | 0.1581 | 50 (28-72) | 74 (49-91) |
